# Barriers and Facilitators of Adherence to Antiretroviral Drug Therapy and Retention in Care among Adult HIV-Positive Patients: A Qualitative Study from Ethiopia

**DOI:** 10.1371/journal.pone.0097353

**Published:** 2014-05-14

**Authors:** Woldesellassie M. Bezabhe, Leanne Chalmers, Luke R. Bereznicki, Gregory M. Peterson, Mekides A. Bimirew, Desalew M. Kassie

**Affiliations:** 1 Division of Pharmacy, School of Medicine, University of Tasmania, Tasmania, Australia; 2 College of Medicine and Health Science, Bahir-Dar University, Gojjam, Ethiopia; 3 Department of Internal Medicine, Felege-Hiwot Hospital, Gojjam, Ethiopia; 4 Department of Internal Medicine, Gondar University Hospital, Gondar, Ethiopia; UCL Institute of Child Health, University College London, United Kingdom

## Abstract

**Background:**

Antiretroviral therapy (ART) has been life saving for hundreds of thousands of Ethiopians. With increased availability of ART in recent years, achievement of optimal adherence and patient retention are becoming the greatest challenges in the management of HIV/AIDS in Ethiopia. However, few studies have explored factors influencing medication adherence to ART and retention in follow-up care among adult Ethiopian HIV-positive patients, especially in the Amhara region of the country, where almost one-third of the country’s ART is prescribed. The aim of this qualitative study was to collect such data from patients and healthcare providers in the Amhara region of Ethiopia.

**Methods:**

Semi-structured interviews were conducted with 24 patients, of whom 11 had been lost to follow-up and were non-persistent with ART. In addition, focus group discussions were performed with 15 ART nurses and 19 case managers. All interviews and focus groups were audio-recorded, transcribed, and coded for themes and patterns in Amharic using a grounded theory approach. The emergent concepts and categories were translated into English.

**Results:**

Economic constraints, perceived stigma and discrimination, fasting, holy water, medication side effects, and dissatisfaction with healthcare services were major reasons for patients being non-adherent and lost to follow-up. Disclosure of HIV status, social support, use of reminder aids, responsibility for raising children, improved health on ART, and receiving education and counseling emerged as facilitators of adherence to ART.

**Conclusions:**

Improving adherence and retention requires integration of enhanced treatment access with improved job and food security. Healthcare providers need to be supported to better equip patients to cope with the issues associated with ART. Development of social policies and cooperation between various agencies are required to facilitate optimal adherence to ART, patient retention, and improved patient outcomes.

## Introduction

Antiretroviral therapy (ART) decreases progression to Acquired Immune Deficiency Syndrome (AIDS) and prolongs, and improves the quality of, life. Over 800,000 patients are living with Human Immunodeficiency Virus (HIV)/AIDS in Ethiopia and the prevalence of HIV/AIDS in the general population is estimated to be 1.5% [1]. In the past 8 years, decentralization and scale-up of the HIV care program have occurred and by the end of 2011, 249,174 adult patients (86% of eligible patients) had been prescribed ART [1].

Adherence to a medication regimen is defined by Cramer et al as “the act of conforming to the recommendations made by the provider with respect to timing, dosage, and frequency of medication taking” [2]. To optimize ART, at least 95% adherence is required in order to prevent the development of resistant viral strains, although regimens with boosted protease inhibitors (PIs) or non-nucleoside reverse transcriptase inhibitors (NNRTIs) can achieve good viral suppression even below this level of adherence [3]. Non-adherence to ART may result in regimen failure, immune suppression, emergence of resistant viral strains, limited future treatment options, and higher treatment costs [4].

Adherence to a medication is a dynamic behaviour influenced by many factors. The findings of several studies conducted in resource-limited settings have shown that major facilitators of ART adherence encompass social support, positive treatment outcomes, and life-long projects [5], [6], [7]. Factors such as cost of medications, access to health facilities, transport costs, and fear of stigma and discrimination are recognized barriers to adherence with ART [8], [9], [10].

Achievement of optimal adherence and patient retention [11] are becoming the greatest challenges in the management of HIV/AIDS in Ethiopia. A five-year retrospective medical record review of 3012 adult patients who were enrolled in therapy at Gondar University Hospital ART clinic demonstrated that 31.4% had been lost to follow-up [12].

To our knowledge, only three qualitative studies have attempted to identify factors that influence adherence to ART in adult patients with HIV/AIDS in the Ethiopian setting. Financial constraints, distance to ART clinics, patient load, patients’ beliefs, and alcohol and drug use were identified as barriers to retention in the Ethiopian healthcare setting [13], [14], [15]. Previous studies have mainly focused on exploring factors influencing patient retention at the healthcare level and were limited in their ability to identify barriers to, and facilitators of, medication adherence at the individual level. Moreover, none of these studies were performed in the Amhara region of Ethiopia, where 31.7% of the country’s ART usage occurs [16]. The region is home to 20 million people, of whom more than 91% are Amhara and about 80% are Orthodox Christians [17]. This study sought to examine the enablers and barriers to medication adherence to ART, including reasons for patients being lost to follow-up, in the Amhara region of Ethiopia.

## Methods

### Study Setting

This study was carried out at Felege-Hiwot Hospital and Gondar University Hospital, Northwest Ethiopia. Each hospital serves a catchment area of 5 million people. In 2005, when free ART was launched in the country, both hospitals began to offer ART. The numbers of patients receiving ART were 4,378 and 6,265, in Gondar University Hospital and Felege-Hiwot Hospital, respectively, at the time of data collection.

Adult patients with HIV-infection and CD4 count less than or equal to 350 cells/mm^3^ regardless of the clinical symptoms, or with any symptoms indicating a WHO clinical stage of 3 or 4, irrespective of CD4 count, are eligible to start ART. At both hospitals, specific ART nurses provide counseling to patients diagnosed as HIV-positive before and after ART initiation, while the physicians working in the ART clinics initiate ART medication based on patients’ clinical and laboratory findings. Nurses are also responsible for assessing patients’ progress at regular appointments in ART clinics, every month for the initial six months and every three months thereafter. If patients have any complaints, such as opportunistic infections (OIs) and drug side effects, the nurses refer patients to the physician to receive treatment. Furthermore, the study clinics have a peer support program staffed with case managers who themselves take ART. ART nurses refer patients to case managers for adherence counseling and management of ART risk factors. Case managers are certified care providers responsible for managing ART risk factors and locating patients lost to follow-up. They carry out a programmed outreach service to track patients who have been lost to follow-up and are not reachable by phone, and to provide onsite community support.

### Inclusion/Exclusion Criteria

Adult patients (≥18 years old) who had been receiving ART in the study clinics for at least a month and could provide informed consent in Amharic were eligible for the interviews. All ART nurses and case managers who had been working in the ART clinics for at least 6 months were invited to participate in the focus group discussions.

### Recruitment and Sampling

ART nurses and case managers aware of patients’ treatment histories, supplemented by checking of patients’ medical records, helped to identify and recruit possible participants into the study. The recruitment of patients continued until information saturation was achieved. Research pharmacists working across the two sites invited all ART nurses and case managers to participate.

### Ethics Statement

The Tasmanian Social Sciences Human Research Ethics Committee, University of Tasmania, Australia, and College of Medicine and Health Science Ethics committee, Bahir-Dar University, Ethiopia, approved this study. Patient interviews were conducted in private rooms where patients felt safe and stress-free. Focus group discussions were conducted in hospital auditoriums. Focus group participants were required to maintain the confidentiality of the identities of other participants and the content of the discussion. Participants were told either to remove themselves from the room or ask the interviewer to move the interview in another direction if they felt distress during the interview. The purpose of the study was explained to all participants using standard information statements, and written informed consent was obtained before participation. At the end of the session, the participants received 150 birr (∼ $ 7 USD) as compensation for their time and transportation costs. Patients who disclosed non-adherence during interview were referred to case managers in ART clinics for possible intervention. Personal or identifying information was not retained within the transcripts.

### Data Collection

Data were collected from 22 February 2013 to 12 July 2013 using semi-structured interview and focus group discussion guides with patients and healthcare providers. The guides were designed to elicit information from the patients’ and healthcare providers’ points of view based on their experience with HIV medication, including factors that facilitate or constrain adherence to HIV medication and reasons for loss to follow-up. Consistent with an inductive approach, guides were updated continuously based on the results of the ongoing data analysis before the next interview or focus group discussion. A trained research pharmacist conducted the interviews and focus groups in the local language, Amharic. Comprehensive notes were taken throughout and after interviews and focus groups, and all sessions were audio-recorded. Participants decided on the times and the locations of the individual interviews. Data were stored in a locked cabinet in locked facilities in the College of Medicine and Health Sciences at the Bahir-Dar University. All electronic records were stored on a secure, password-protected, server at the University of Tasmania. Researchers were able access the data whenever required. Data will be destroyed 5 years following publication. Paper data will be securely shredded and electronic data will be erased at this time.

### Data Preparation

Upon completion of each focus group and interview, the research pharmacist produced a complete transcript in Amharic. The transcribed data were read and reviewed to ensure understanding and then compared with the original audio-records for accuracy. The study data were established from the transcripts.

### Data Analysis

**Figure 1:**
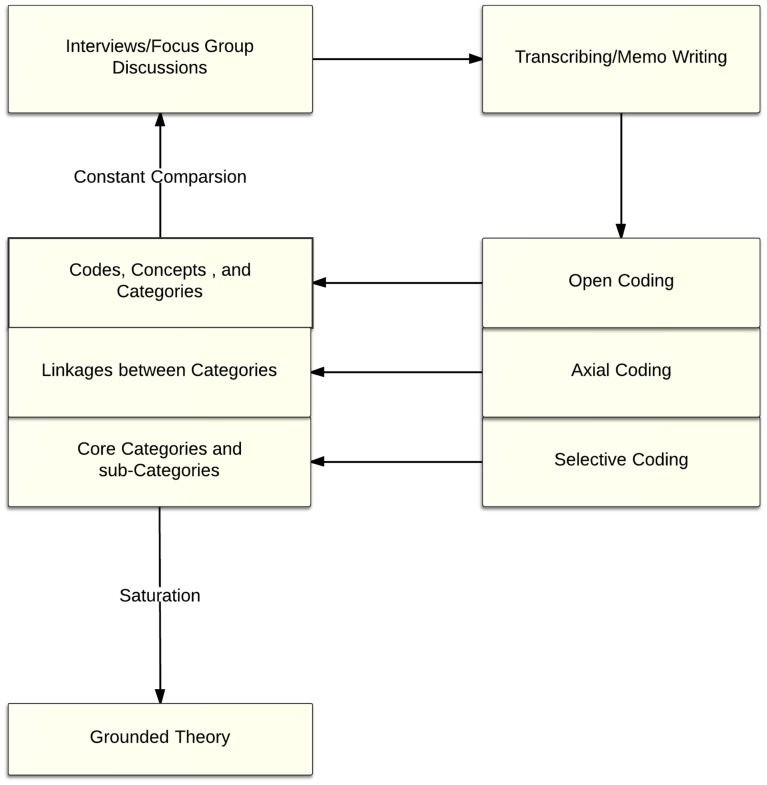
Data Analysis in Generating Grounded Theory.

Analysis of data aimed to describe the barriers to, and facilitators of, adherence to ART including the possible reasons of patients for missing appointments and/or being lost to follow-up at ART clinics. A grounded theory approach was used for analysing the data [18]. The process of data analysis was as shown in figure 1. The documents in Amharic were uploaded into NVivo (QSR, Cambridge, MA) and textual data were coded into themes inductively. Two coders (the research pharmacist and a general practitioner) performed the coding separately and differences were resolved with discussion. Focus groups and interviews were coded and analysed separately, and matched for common themes. The key findings were illustrated by selecting representative quotes.

Two bilingual translators translated emerging concepts and categories into English. The two translators discussed their differences and developed a single English version. A third person translated the final English version back into Amharic. A committee, consisting of an expert in English, a pharmacologist, an expert qualitative researcher and the research pharmacist, settled issues of conceptual equivalence and use of words between concepts and categories in Amharic and the final English version.

## Results

### Study Participants

Twenty-four patients, 15 nurses, and 19 case managers participated in this study. Forty-four face-to-face interviews and 6 focus group discussion sessions were conducted across the two sites.

### Characteristics of Study Participants

#### Patients

The mean age of the patients was 36 years. Half of those who participated were women. Less than half (41%) were married or living with a partner, and 58% had primary school or no education. Approximately half (46%) had been lost to follow-up and returned to the ART clinics either through tracking or on their own. The remaining patients were on follow-up; of these, 69% never missed appointments and 31% missed appointments frequently.

#### Healthcare Providers

Approximately two-thirds (65%) of healthcare providers were women. The mean age of the healthcare providers was 32 years. Forty-four percent were nurses and the rest were case managers.

## Themes and Categories

The themes that emerged were classified as patient-related, healthcare service-related, and medication-related. Each theme was classified into categories as presented below and shown in table 1.

**Table 1:** List of emergent themes that influence adherence to ART and retention in follow-up care.

Themes	Adherence facilitators	Adherence barriers	Factors influencing loss to follow-up
**Theme I: Patient-Related**			
• Economic constraints		✓	✓
• Disclosure and social support	✓		
• Reminders	✓		
• Stigma and discrimination		✓	✓
• Responsibility for raising children	✓		
• Religious rituals			
 Fasting		✓	
 Being baptized with holy water			✓
• Belief of being cured from HIV/AIDS			✓
**Theme II: Healthcare Service-Related**			
• Counselling and education	✓		
• Fatigue of healthcare providers		✓	
• Poor medical record handling		✓	
• Unavailability of office		✓	
• No service at weekends		✓	
**Theme III: Medication-Related**			
• Experiencing improved health on ART	✓		
• Adverse effects		✓	✓

### Theme I: Patient-Related Factors

Patient-related factors such as economic constraints, disclosure of HIV status, social support, use of reminder tools, stigma and discrimination, responsibility to raise children, and religious rituals were the most significant determinants of adherence and loss to follow-up.

### Category I: Economic Constraints

Individual interviews and focus groups indicated that movement for seeking employment and food insecurity impaired adherence to ART amongst patients with HIV/AIDS who had started ART. Almost all patients (11 of 12) who had been lost to follow-up were unemployed, daily laborers or waiters working at a low wage rate. They moved from one part of the country to another part of the country searching for a job. Lack of money for transport, unavailability of nearby ART clinic services, and language and communication barriers prevented such patients from remaining in care.

*I went to Humera to find a job. I got one time a refill from Humera Hospital. After a while, I crossed the Sudan border with friends. I worked in the farm in a jungle for 2 months. I do not speak the Arabic language (so) I was not able to refill my medicine. On the way back to Ethiopia with friends, looters took all (my) money including (my) ART user identification card. Because of this, I missed my medication for about three months. (26 years, male, patient, casual worker, 008)*

*To get a job, patients usually move to remote areas where they cannot get access to ART. For instance, those who went to Sudan did not get the treatment there and were lost to follow-up for long period of time. (28 years, female, nurse, FG5)*

Patients with HIV/AIDS reported reduced physical strength, especially in the initial phase of ART, and were only able to tolerate reduced work hours. Some patients generated a minimal monthly income not sufficient to cover the costs of their basic needs, including food. Thus, unavailability of food hindered patients from taking their HIV medications. Some of the patients, being dependent on the food supply from ART clinics, left their pill bottles with the nurses when they did not receive their food ration from the clinic. Some others patients believed that they had to consume costly food items to take their HIV medications.

*Previously, I was getting (an) additional nutrition supply, plumpy nut, for free from the ART clinic. But the other day, the nurse told me that she would not give it to me anymore. When I heard this, I felt angry and left the medication bottle on the nurse’s desk and went away from the clinic. Due to this I did not take pills for five days. (33 years, male, patient, jobless, 007)*

*When Non-Governmental Organizations (NGOs) stopped food donations, patients complain about continuing ART. Some of them are even lost to follow-up because of the unavailability of food. (32 years, female, case manager, FG3).*

*Most people do not have an idea that the medication can be taken eating any food available. They do not know that they can prepare the available food in the house with proper hygiene. They think the medicine will be harmful if they do not eat an expensive diet such as meat and eggs. (30 years, female, case manager, FG3)*

### Category II: Disclosure of HIV Status and Social Support

Both interview and focus group participants most frequently cited that disclosure of their HIV status to friends, family and neighbors was a facilitator of adherence. Patients who disclosed their HIV status to relatives and close friends did not fear stigma and discrimination to obtain and take drugs.

*I have no problem of disclosing my (HIV) status to my family, friends, and others. When I usually introduce myself, I often tell them that I am living with HIV… this helped me to take the pills regularly, without any anxiety, and to recover from my illness as well. (35 years, female, patient, librarian, 015)*

*Many patients told me that disclosure of their status to neighbors and community helped them to take pills properly. (26 years, female, nurse, FG4)*

Disclosing one’s HIV status was found to be essential for receiving social support. Reminders to take pills, cover for transportation and food costs, and emotional backing were commonly reported. The most important way that patients received support was in reminders regarding the time they took pills. Patients received support usually from their partner, who himself/herself was also on ART, and/or children who were living with them. Experienced ART nurses and case managers noticed that transport and food support from NGOs had improved patients’ attendance at ART clinics.

*Sometimes when I feel fatigued, am busy with work or sleep at dose time my children remind me to take the pills. They bring me a glass of water and the medication bottle. (36 years, female, patient, casual worker, 004)*

*Aids from NGOs such as getting cooking oil, wheat, and soap until HIV patients recover from their illness and support themselves is one facilitator for adherence to ART. (46 years, male, case manager, FG1)*

### Category III: Stigma and Discrimination

Patients and healthcare providers frequently mentioned that stigma and discrimination caused patients trouble in taking, obtaining, and keeping medications. Patients described how they would avoid taking pills in front of others, including family members who did not know their HIV status.

*I used to miss pills many times… because people were with me. The guy with me did not know my HIV status. I worry what people think about me if they know about my status while I am taking pills in front of them. (25 years, female, patient, waitress, 020)*

*Only my mother knows about my HIV status… I fear stigma and discrimination if other family members noticed me taking the pills. (20 years, female, patient, waitress, 006)*

Patients also preferred to attend treatment in clinics far away from home to avoid disclosure of their HIV status to their community members.

*Fearing disclosure in their residential area…. patients attend ART from Gondar when they have access to it in Bahir-Dar and vice versa. That creates a challenge for patients in picking up their medicine later. (23 years, male, nurse, FG1)*

Lack of privacy was a particular challenge for patients working in a private home or organization. Half of the patients were part-time workers, daily laborers or waiters. These patients were forced to work at a low wage rate for long hours to cover their living costs in places where their privacy could not be maintained. They had problems with finding a place to keep and take their medications; they thought that they had to hide their pills from employers and colleagues. Patients felt that they might be dismissed from their job if employers learned of their status.

*If the employer learns that I am living with HIV… I fear to be sacked from my job. (26 years, female, patient, casual worker, 019)*

*When I asked my patient, how she was taking her pills? Whether she kept her time or not… she told me that she is a daily laborer baking injera and abshilo in other people’s houses. If employers learn that she is living with HIV and taking the pills they would stop her working with them. She used to take pills only when they went away from her and she was alone. As the result she does not take her pills on time. (45 years, female, nurse, FG5)*

*I was working as a cooker in a cement factory compound for Indian employees. For some time, I was taking the pills hiding myself (so as) not to be noticed by anyone. But, later, I became worried thinking that if they knew my status, they might dismiss me from my job. Then I stopped taking the pills. (27 years, female, patient, cohabiting, 018)*

### Category IV: Responsibility for Raising Children

Patients’ commitment to raise and educate their children facilitated medication taking. Patients with HIV were at the peak of their reproductive lives; 14 out of 24 (mean age 36) had dependent children. They fear passing away with AIDS, leaving their children as orphans. Of 14 patients who were living with their children, 9 were found to be adherent by their ART nurses.

*… I have a boy born free of the virus. As long as I am alive, I wish to see him achieving better opportunities. If I am not taking the medication properly or abandoned it altogether, I am ruining his chance. (35 years, male, patient, self-employed, 002)*

*Patients usually say that if they are not alive, it is difficult to imagine the survival of their children. They told me that the medicine kept them healthy to work and help their children to achieve a better future. (35 years, male, nurse, FG5)*

### Category V: Reminders

Patients frequently mentioned that setting alarms on watches or mobile phones helped them to remember to take their pills. Seven respondents said they set alarms on their mobile phones or watches. Providers also stressed the benefit of reminders in avoiding pill missing due to hectic daily activity.

*To take my pills on time, I set a reminder on my mobile phone…it reminds me of the time of my medication even when I forget it. (41 years, male, patient, casual worker, 016)*

*Especially use of mobile phones or watch alarm tones is more sustainable for not forgetting taking pills. (27 years, female, case manager, FG6)*

Although electronic reminder tools have the aforementioned advantages, some patients did not have their own mobile phone or watch, and some others were too illiterate to use these reminder tools. Eight respondents had no formal education and were not able to write and read. These patients used other alternative reminders such as the position of the sun, length of shadows, entrance and exit time of domestic animals and students, and bells from a church or mosque or factories to take their pills on time. Although such kinds of devices were helpful as pill reminders, they were limited in their ability to indicate the exact time.

*I take pills whenever I hear Allah Akbar. (26 years, male, patient, casual worker, 008)*

*I have an old lady customer who is too illiterate to read a watch and who has no family or relatives living with her…she usually takes her pills looking at students going to school in the morning. (33 years, male, nurse, FG5)*

### Category VI: Religious Rituals

Religious rituals like fasting and holy water were found to influence medication taking. Both Ethiopian Orthodox Christian followers and Muslims have several fasting days and seasons within a year.

#### Sub-category I: Fasting

During fasting seasons, some patients did not take their medications properly.

*I take the morning dose at midday during fasting since I have to wait without any meal until midday. (38 years, female, patient, secretary, 023)*

*(Orthodox Christian) patients have a strong attitude towards fasting and become late in taking pills on Friday and Wednesday or in any fasting season. Patients take the morning dose at 12 am. (52 years, male, case manager, FG3)*

*During Ramadan, I only take the evening dose. It is impossible to take the morning dose as we eat during the nighttime. (38 years, male, patient, driver, 024)*

*During Ramadan some Muslims do not take the daytime dose. They only take the evening dose while taking their food. They usually come and consult us on what to do. (40 years, female, nurse, FG2)*

#### Sub-category II: Being Baptized with Holy Water

Going to monasteries to be baptized with holy water was the most frequently mentioned reason for patients being lost to follow-up. This was reported by five of the 11 patients lost to follow-up; all of them were females. Some Orthodox priests preached that patients should take holy water, pray to God, and stop taking their pills to be cured of their ‘curse’.

*I stopped taking my medication while I started being baptized with holy water (in a monastery). Priests there told us only to be baptized by the holy water and stop taking antiretroviral pills; I believed in Jesus and took only the holy water to be cured. (20 years, female, patient, waitress, 006)*

Patients preferred complete cure from HIV/AIDS with the holy water treatment rather than taking pills throughout their life. Interviewees and focus group members reported discontinuation of ART treatment in those being baptized with holy water.

*I have friends who were living with HIV; they told me that they get cured after attending holy water treatment for three months in a monastery. They told me to go and get the treatment; in addition I have been informed to stop the pills citing that I do not have to believe in the two things simultaneously to be cured. I left my pills at home and went there for two months. (24 years, female, patient, waitress, 022)*

*During the time when I was tracking lost patients, members of a lost patient’s family told me that she is cured from her disease after she had been baptized from a monastery holy water. They tried to convince me to go and check her health status. Still now she has not come back to the clinic. (31 years, female, case manager, FG2)*

*Some of the patients were lost to follow-up after they were baptized by the holy water; they told us that they are cured from HIV/AIDS because of the holy water. (36 years, male, nurse, FG5)*

### Theme II: Healthcare-Related Factors

Healthcare-related factors, such as patient education and counseling facilitated medication adherence, while busyness of healthcare providers, poor laboratory service, and poor medical record handling impaired adherence and retention.

### Category I: Counseling and Education

Education and counseling of patients in the ART clinics motivated them to take their pills. Case managers and ART nurses provided education, which focused on the importance of perfect adherence, strategies to improve adherence, consequences of non-adherence, possible side effects of the medications, and the duration of treatment required.

*Every morning in the waiting room nurses and case managers teach us the consequences of not taking pills properly and the benefits of disclosure to family and the community. That helps me a lot to adhere to the treatment and improves my health condition. (36 years, female, patient, self-employed, 014)*

*If the healthcare provider who initiated ART gives comprehensive education, including how long the medications will be taken, the possible side effects of the medications, the importance of the treatment, whether it cures or not, and checks patients’ understanding at the end, it will help patients to continue their treatment without interruption. (28 years, female, nurse, FG5)*

### Category II: Fatigue of Healthcare Providers

In contrast, healthcare providers were fatigued with a high load of patients; this impaired the quality of the service delivered. Sometimes, it was felt that ART nurses did not have empathy and rushed when writing prescriptions, without addressing patients’ concerns about their treatment. This impaired the ART nurse-patient relationship and patients did not enjoy coming to the clinics.

*They abuse you, they do not accept you in good terms, and I feel bad about these (things). Even, I feel my illness more when I come to the clinic and see their faces. I feel as if I went to a police station and was interrogated as a criminal. (38 years, female, patient, secretary, 023)*

*…The hospital is crowded … it has many patients… professionals are fatigued with it. (52 years, male, case manager, FG3)*

### Category III: Poor Laboratory Service

The ART clinics have large numbers of patients; there are queues to receive services. Patients were instructed to come to ART clinics early in the morning and line up to have their CD4 counts checked. Some of them were too busy to do so and unable to attend to their treatment properly.

*I came (to the ART clinic) early in the morning at 5 am to get a CD4 test. We were forced to struggle with the coldness and thieves at this time. This is the reason why I did not check my CD4 count regularly and attend my treatment properly. (38 years, female, patient, secretary, 023)*

*The result of CD4 count tests are delayed up to one day after the test; this needs to be corrected. (28 years, female, case manager, FG6)*

### Category IV: Poor Medical Record Handling

Several patients and healthcare providers mentioned that there have been serious problems in handling of patients’ medical records in the hospitals. Many patients experienced loss of their medical record and were forced to start a new medical record, losing all their previous data. Sometimes patients were forced to repeat CD4 count measures when the laboratory report was lost from the medical record and was therefore unavailable to inform medical decisions.

*My chart was lost from the chart room…the nurse told me to I needed a new chart but I was not voluntary to have a new one…I left the clinic citing the need for my previous documents for prescribing of my medications. (35 years, male, patient, self-employed, 002)*

*There is problem of chart loss. Sometimes you find the empty cover of the chart while all the documents are lost. Patients were forced to repeat CD4 tests as a result. (36 years, female, case manager, FG2)*

### Theme III: Medication-Related Factors

Improvements of health on ART and medication side effects were the two consistent and contrasting themes related to medication. Improvement of health on ART facilitated adherence to HIV medication, while medication side effects was among the reasons for patients to be non-adherent or lost to follow-up.

### Category I: Improved Health on ART

Most patients had been confirmed as HIV-positive after suffering a long-term sickness. They had seen the devastating effect of HIV on their bodies and had vivid illnesses and stories. A significant improvement of health witnessed soon after initiating ART heightened trust in the medications.

Both patients and providers highlighted being healthy, improved appetite, increased weight and CD4 count, and prolonged lives as positive treatment outcomes. Patients were no longer bedridden, had strength, resumed work, and increased their incomes. These improvements inspired them to continue taking their medicines.

*I have decided to be more committed towards the treatment because I have seen the benefits of antiretrovirals. My appetite has improved, I was bedridden, but now I am healthy and I usually find myself at the field for work…. I achieved all this because of the pills. Hence, I should not stop taking the pills. (35 years, male, patient, farmer, 016)*

*… Improvement of health after treatment of OIs, being able to live longer and progressive increments of CD4 counts were the major factors strengthening medication taking. (24 years, male, nurse, FG5)*

### Category II: Adverse Effects

Patients who experienced unanticipated and/or intolerable side effects, like nightmares and psychosis, missed doses or discontinued therapy altogether. This was particularly likely among patients who had started ART when asymptomatic as they considered the medications had worsened their health.

*I was not conscious (after taking the medications), I was mad, as neighbors told me. I went to a neighbor’s house and shouted, calling on a certain woman, blaming her for being evil eye and making me sick. After that I stopped taking the pills. (26 years, female, patient, casual worker, 019)*

*When I phoned a lost patient who was on TDF/3TC/EFV, for tracking, he told me that he could not come back unless the regimen changed. That was because he had developed nightmares due to the efavirenz. (33 years, male, case manager, FG2)*

*At the beginning of my treatment, I experienced vomiting. As the result, I missed some doses, until I adapted to the medication. (30 years, female, patient, waitress, 009)*

## Discussion

We sought to understand the barriers and facilitators that influence adherence in patients taking ART in the Amhara region of Ethiopia. We identified economic constraints as the greatest barrier, with fear of stigma and discrimination, fasting and holy water, poor healthcare services, and medication side effects also interfering. Meanwhile, disclosure of HIV status, social support, use of reminders, life-long projects, counseling and education, and improved health on ART facilitated medication adherence and retention in HIV care. Most of these factors were consistent with the findings reported in other regions of Ethiopia [13], [14], [15] and elsewhere in resource-limited settings [19], [20], [21]. However, some of the findings such as migration [22], [23], [24], [25], [26], holy water, fasting [19], and traditional time reminders have been rarely reported or not reported at all in other settings. These differences may be attributed to the socio-economic and socio-cultural differences of our study sample. The major findings that had significant impact on adherence including economic constraints, stigma and discrimination, and disclosure of HIV status and the new or the rarely reported findings highlighted above are discussed below.

The two major socio-economic constraints that negatively affected adherence and retention in HIV care in our study were a lack of work, resulting in the need for migration to find a job, and food insecurity. These factors have also been documented in other studies [8], [19], [22], [23], [25], [26], [27], [28]. Patients were lost to follow-up when they migrated to other places either inside or outside Ethiopia to find a job. Migration of Ethiopian youths in large numbers from rural to urban areas of the country to find work, as well as out of the country to the Middle East and sub-Saharan African countries, has been reported by other authors [29], [30]. Patients also missed pills and stopped collecting repeat prescriptions from clinics when they could not afford to buy food, or when NGOs stopped supplying food rations. The negative impact of food insecurity on adherence has also been recognized in other studies conducted in sub-Saharan Africa [8], [27], [28]. Lack of job opportunities and food insecurity, while not exceptional to patients with HIV/AIDS, were exacerbated by the co-existence of other HIV-associated challenges such as stigma and discrimination, reduced physical activity, medication schedules, and indirect treatment costs. Proactive strategies to improve access to jobs and food security for patients taking ART are required in Ethiopia. Both governmental and non-governmental organizations need to work in coordination to address the multilayered disadvantages in patients receiving ART.

Socio-cultural factors, such as stigma and discrimination and religious rituals, also had undesirable effects on medication adherence and remaining in care in this sample. Stigmatization and discrimination are complex socio-cultural phenomena that arise from the perception that a person with HIV/AIDS has unwanted qualities, thus reducing him in the eye of society [31]. Perceived stigma (felt stigma) and discrimination and lack of privacy to take and collect medication hindered adherence to ART in this study; these factors were also identified in other sub-Saharan and Far-East Asian studies [19], [32], [33], [34], [35], [36]. As reported by other authors [26], patients in this study had concerns about being stigmatized and losing their jobs if their HIV status was discovered when they took pills in front of work colleagues or asked permission from their employers to collect medications at appointment dates. Seeking treatment at health facilities far away from home to hide their HIV status from family and colleagues is consistent with findings of other qualitative studies [19], [37].

A multi-faceted intervention, including information provision, skill building, counselling and facilitating interaction between people with HIV/AIDS and the community, has the potential to reduce stigma and improve ART adherence and retention in care [38], [39], [40]. A systematic review by Stangl et al. reported that there was a lack of effective stigma reduction strategies that could be implemented on a large scale [38]. Tackling stigma and discrimination at multiple levels might also improve patients' abilities to adhere to ART treatment and continue presenting for medical care in Ethiopia.

Religious beliefs are complex cultural concepts and influenced patients’ treatment with antiretrovirals in our sample of study; this has been reported elsewhere [19], [41], [42]. The Ethiopian Orthodox Church does not allow eating until midday every Wednesday and Friday, as well as in fasting seasons such as Flseta and Worha-Tsom. Similarly, Muslims do not eat during the daytime in Ramadan. Patients miss or delay medications to fulfill these religious obligations. Going to monasteries to be baptized with holy water was found to be the most important reason for patients being lost to follow-up in this study. Another study in Ethiopia has also found seeking traditional treatment and/or holy water treatment to be the most important reason for patients being lost to follow-up care [43]. Stakeholders, governmental and non-governmental, should work with religious authorities to reduce the negative impact of fasting and holy water on medication-taking.

Disclosure of HIV status, social support, and use of reminders were identified as important facilitators of adherence in this study; other studies have reported similar findings [19], [44], [45], [46], [47], [48], [49], [50]. Case managers and ART nurses encouraged patients to disclose their HIV status to family members or close friends who can provide them social support, reminders about pills, financial assistance, and emotional backing to facilitate medication taking in our study and elsewhere [7], [20], [51], [52]. Case managers at the clinics are also working to deliver emotional support and resolve patients’ problem based on their experiences living with HIV. Intervention studies reported that integration of dialogue and thinking about what needs to be done prior to disclosure, role-playing, and behavioral exercise were more effective than separate interventions in promoting disclosure [53], [54]. There appears to be a role for these types of interventions to enhance disclosure of HIV status and therefore aid in the achievement of optimal adherence and retention in care in Ethiopian patients taking ART as well.

One facilitator of medication adherence was the use of electronic devices, such as mobile phones and alarms. These have the advantage of reminding patients of their medication times without the need for disclosure of their HIV status to others. Access to mobile phones is increasing in Ethiopia [55] and setting alarm tones on mobile phones helped patients to remember to take pills. Randomized controlled trials in Kenya reported a mobile phone short message service improved adherence to ART treatment and retention in medical care [49], [50]. Healthcare providers need to use the opportunity of increasing access to mobile phones in the local area [55] for improving patients’ adherence to HIV medication. Illiterate patients used the position of the sun, entrance and exit time of students, and the bell or sound of prayer time as their reminder to take pills. This finding is unique; no other studies have reported the use of the aforementioned traditional ways of time measuring for taking pills. Given many Ethiopian HIV-positive patients are illiterate [56] and depend on traditional ways of time counting, which do not measure point to point medication time and influenced by many factors, the healthcare providers need to train patients how to use the simple electronic reminder devices to improve adherence.

## Strengths and Limitations

One of the primary strengths of this study was the use of multiple data sources including focus group discussions and semi-structured interviews, involving patients on ART, ART nurses, and case managers across two sites. Almost all participants accepted our offer and were involved in the study; the non-response rate was very low. This may have been due to scarcity of this kind of research in our region of Ethiopia, and thus this research gave an opportunity for participants to share their experiences of ART. Both patients, who were adherent and non-adherent, including those who had been lost to follow-up from ART clinics, were included in this study.

The study has some limitations. People who were picking up medications for someone else were not included in this study. Moreover, patients who were bedridden or with psychiatric or other problems who were not able to attend ART clinics at the times of data collection were not interviewed.

## Conclusion

Scale-up of treatment and care for patients with HIV/AIDS in sub-Saharan Africa has been a decisive clinical achievement. The full benefit of the scale-up cannot be realized without achieving long-term optimal adherence and retention in care. In this study we found that economic constraints, perceived stigma and discrimination, fasting, holy water, and poor healthcare services hamper adherence to ART and retention in care. Conversely, disclosure of HIV status, social support, use of reminder aids, having life-long projects, and patient education and counseling facilitated adherence and retention in care. International studies have demonstrated that interventions directed at many of these factors have encouraged patients to achieve optimal adherence and remain in care. Interventions integrating enhanced treatment access with improved job and food security, supporting healthcare providers, development of social policies, and cooperation between various agencies are required to facilitate optimal adherence to ART, retention in care, and improved patient outcomes.
